# From Refractory Iron Deficiency Anemia to the Diagnosis of Hereditary Hemorrhagic Telangiectasia Type 1: A Case Report

**DOI:** 10.7759/cureus.117613

**Published:** 2026-10-07

**Authors:** Mahendra Dhaka, Nidhi Nidhi, Arjun Kachhwaha, Taruna Yadav, Gopal K Bohra

**Affiliations:** 1 Department of General Medicine, All India Institute of Medical Sciences, Jodhpur, IND; 2 Department of Clinical Hematology, All India Institute of Medical Sciences, Jodhpur, IND; 3 Department of Diagnostic and Interventional Radiology, All India Institute of Medical Sciences, Jodhpur, IND; 4 Department of Radiology, All India Institute of Medical Sciences, Jodhpur, IND; 5 Department of Medicine, All India Institute of Medical Sciences, Jodhpur, IND

**Keywords:** bevacizumab, endoglin, hereditary hemorrhagic telangiectasia, iron deficiency anemia, osler-weber-rendu disease, pulmonary arteriovenous malformation

## Abstract

Hereditary hemorrhagic telangiectasia (HHT) is an autosomal-dominant vascular disorder characterized by mucocutaneous telangiectasias and visceral arteriovenous malformations (AVMs) resulting in recurrent epistaxis, gastrointestinal bleeding, iron deficiency anemia, and complications related to pulmonary and cerebral vascular shunting. We report the case of a 70-year-old man with a 20-year history of refractory iron deficiency anemia requiring frequent blood transfusions and intravenous iron therapy who presented with recurrent epistaxis, melena, exertional dyspnea, and bilateral lower-limb edema. Examination demonstrated multiple telangiectasias involving the fingertips, nasal mucosa, oral cavity, and tongue. Endoscopic evaluation revealed multiple bleeding telangiectatic lesions involving the stomach, jejunum, and colon, with argon plasma coagulation performed for hemostasis. Contrast-enhanced computed tomography demonstrated multisystem AVMs involving the lungs, liver, and spleen, while no cerebral AVM was identified. Contrast echocardiography demonstrated a delayed appearance of microbubbles in the left atrium after five cardiac cycles, consistent with an intrapulmonary shunt. Genetic testing identified a pathogenic variant in the endoglin (ENG) gene, confirming HHT type 1. A significant pulmonary arteriovenous malformation was treated with transcatheter embolization. In view of severe recurrent bleeding and transfusion-dependent anemia despite local measures, systemic bevacizumab was initiated at 5 mg/kg every two weeks for six doses, followed by monthly maintenance therapy. The patient subsequently demonstrated a marked clinical response, with improvement in hemoglobin levels and cessation of melena, without an infusion-related adverse reaction. This case highlights the importance of recognizing HHT in patients with longstanding unexplained iron deficiency anemia and recurrent bleeding from multiple vascular territories and demonstrates the potential role of systemic bevacizumab in patients with severe HHT-associated bleeding.

## Introduction

Hereditary hemorrhagic telangiectasia (HHT), also known as Osler-Weber-Rendu disease, is an autosomal dominant vascular disorder characterized by mucocutaneous telangiectasias and visceral arteriovenous malformations (AVMs). The disease most commonly involves the nasal mucosa, gastrointestinal tract, lungs, liver, and brain and may result in recurrent bleeding, severe iron deficiency anemia, high-output cardiac failure, hypoxemia, paradoxical embolic events, and neurologic complications [1,2].

The diagnosis can be established clinically using the Curaçao criteria, which include recurrent spontaneous epistaxis, multiple characteristic telangiectasias, visceral AVMs, and a first-degree relative with HHT. The presence of three or more criteria establishes a definite clinical diagnosis. Molecular identification of a pathogenic variant in an HHT-associated gene provides additional diagnostic confirmation, particularly in patients with an absent or uninformative family history [1,2].

The most frequently implicated genes are *ENG*, encoding endoglin and associated predominantly with HHT type 1, and *ACVRL1*, encoding activin receptor-like kinase 1 and associated predominantly with HHT type 2. Pulmonary AVMs are particularly frequent among patients with pathogenic *ENG* variants [3].

Management of HHT is multidisciplinary and depends on the affected vascular beds and severity of bleeding. Local treatment of epistaxis and endoscopic ablation of gastrointestinal telangiectasias are important components of management. Pulmonary AVMs with significant feeding vessels should be treated with transcatheter embolization to reduce the risk of paradoxical embolic and infectious complications [1,2]. Iron replacement and blood transfusion remain essential for patients with severe anemia. However, some patients can develop persistent transfusion-dependent bleeding despite repeated local interventions.

Systemic antiangiogenic therapy with intravenous bevacizumab has emerged as an important treatment option for moderate-to-severe HHT-associated bleeding. Large multicenter observational data have demonstrated improvements in hemoglobin concentration, epistaxis severity, red blood cell requirements, and intravenous iron requirements following treatment [4]. We present a patient with genetically confirmed HHT type 1, multisystem AVMs, transfusion-dependent iron deficiency anemia, gastrointestinal bleeding, and recurrent epistaxis who achieved substantial clinical improvement following multimodal treatment, including systemic bevacizumab.

## Case presentation

A 70-year-old man with no known comorbidities presented with progressively worsening exertional dyspnea, bilateral lower-limb swelling, recurrent melena, and intermittent spontaneous epistaxis for the last 20 years. His symptoms had worsened over approximately six months before presentation.

The patient had a remarkable history of refractory iron deficiency anemia for approximately 20 years. During this period, he had required repeated packed red blood cell (PRBC) transfusions and multiple courses of intravenous ferric carboxymaltose (FCM). Immediately prior to the current evaluation, his bleeding severity had increased substantially, and he required PRBC transfusion approximately every 10 days. There was no reported family history suggestive of similar illness. On examination, the patient was hemodynamically stable. He had clinically evident pallor and bilateral pedal edema. Multiple small telangiectatic lesions were identified over the fingertips, nasal mucosa, oral mucosa, and tongue (Figure 1). 

**Figure 1:**
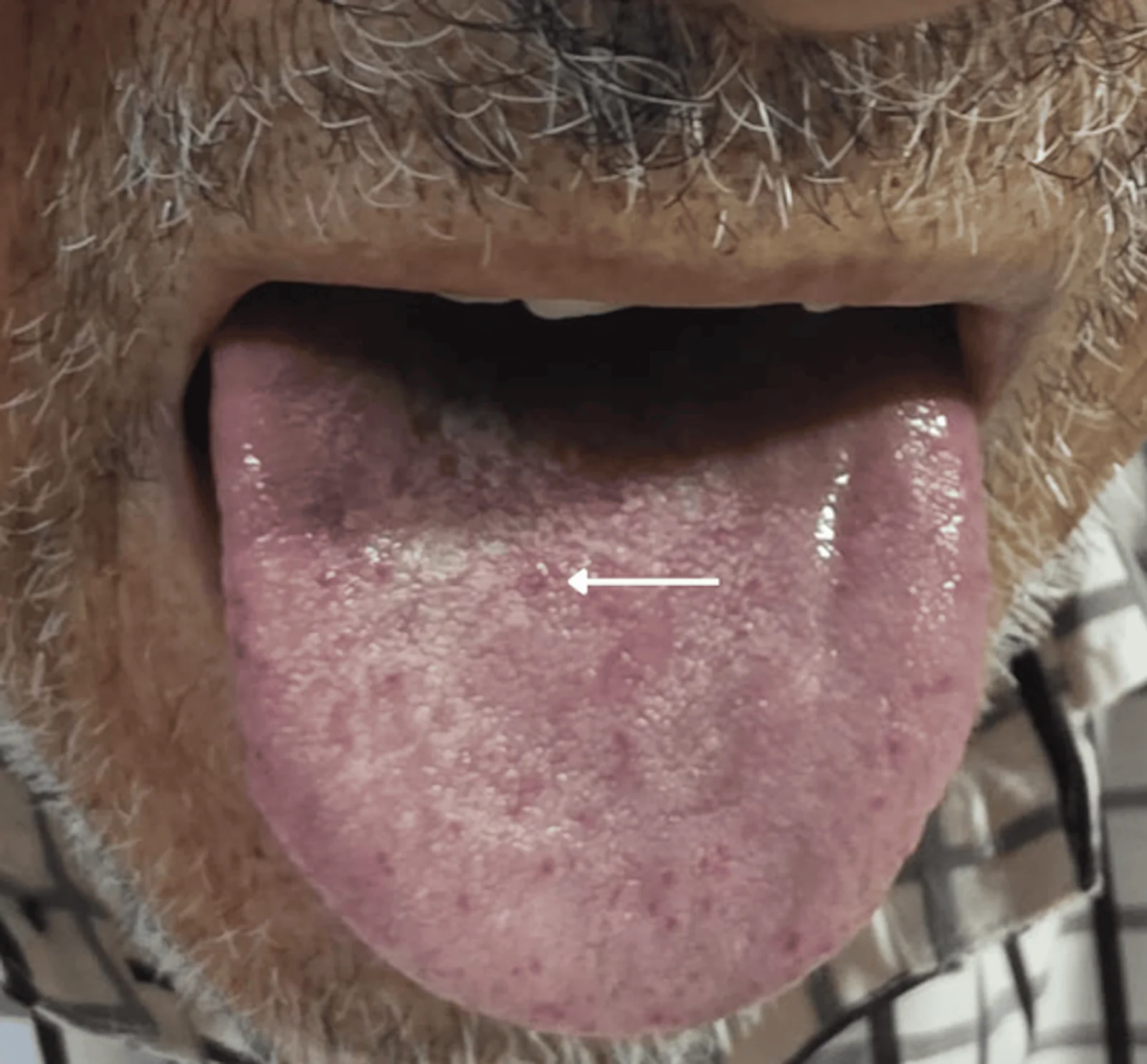
Lingual telangiectasias in hereditary hemorrhagic telangiectasia. Clinical photograph demonstrating multiple small, punctate telangiectatic lesions over the dorsal surface of the tongue (arrow).

Laboratory investigations demonstrated severe iron deficiency anemia. Stool occult blood testing was positive. The coagulation profile and other routine laboratory investigations were within normal limits (Table 1).

**Table 1:** Complete Laboratory Profile Abbreviations: Hb, hemoglobin; MCV, mean corpuscular volume; TLC, total leukocyte count; PLT, platelet count; TIBC, total iron-binding capacity; PT, prothrombin time; INR, international normalized ratio; APTT, activated partial thromboplastin time; SOBT, stool occult blood test.

Investigation	Day 1	Day 90 (Post-Bevacizumab)	Normal Range	Unit
Hemoglobin (Hb)	4.1	13.1	13–17	g/dL
MCV	59	88	80–100	fL
TLC	4.65	4.50	4–11	×10³/µL
PLT	284	340	150–450	×10³/µL
Reticulocyte count	1.2	3.5	0.5–2.5	%
Vitamin B12	450	390	200–900	pg/mL
Serum Iron	11	80	65–175	µg/dL
TIBC	400	260	250–450	µg/dL
Ferritin	5	80	30–400	ng/mL
PT	13	13	11–14	sec
INR	1.1	0.9	0.8–1.2	—
APTT	27	27	26–30	—
SOBT	Positive	Negative	—	—

Upper gastrointestinal endoscopy was performed because of recurrent melena and demonstrated multiple telangiectatic lesions with evidence of bleeding (Figure 2). Argon plasma coagulation (APC) was performed for hemostasis. Subsequent gastrointestinal evaluation demonstrated additional telangiectatic lesions involving the jejunum and colon.

**Figure 2:**
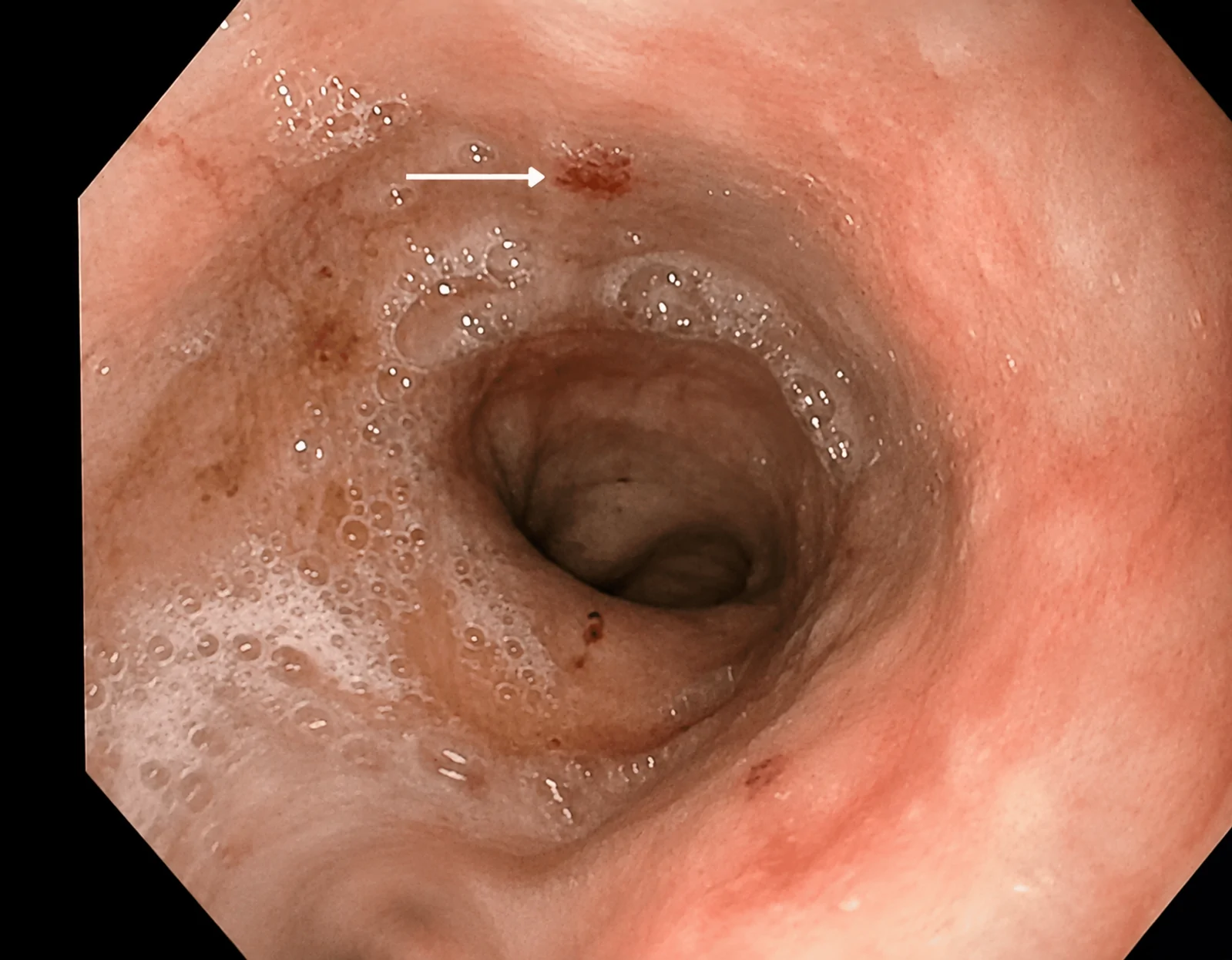
Gastrointestinal telangiectasia in hereditary hemorrhagic telangiectasia. Upper GI endoscopic image demonstrating a mucosal telangiectatic vascular lesion (arrow) in the stomach.

An otorhinolaryngology consultation was obtained for recurrent epistaxis. Nasal examination demonstrated multiple telangiectasias involving both nasal cavities. Chemical cauterization was performed, followed by temporary nasal packing. The nasal packing was subsequently removed during follow-up without complications. The patient's Epistaxis Severity Score (ESS) was 5.7, indicating a clinically significant epistaxis burden. Contrast echocardiography demonstrated a delayed appearance of microbubbles in the left atrium after approximately five cardiac cycles following right-sided chamber opacification. This finding was consistent with an intrapulmonary right-to-left shunt and raised suspicion for pulmonary arteriovenous malformation (PAVM).

Computed tomography angiography subsequently demonstrated multiple visceral vascular abnormalities, including pulmonary AVMs, hepatic AVMs, and splenic AVMs. The liver and spleen were enlarged, with prominent dilated and tortuous hepatic and splenic arterial vasculature (Figure 3). Contrast-enhanced MRI of the brain was subsequently performed for cerebral AVM screening and did not demonstrate any cerebral AVM. Given the presence of PAVM, the patient underwent prophylactic transcatheter embolization of the clinically significant pulmonary AVM. The patient fulfilled three of the four Curaçao criteria for HHT - recurrent spontaneous epistaxis, characteristic mucocutaneous telangiectasias involving the fingertips, nasal and oral mucosa, and tongue, and visceral vascular malformations including pulmonary, hepatic, and splenic AVMs with gastrointestinal telangiectasias causing recurrent bleeding. There was no family history suggestive of HHT. Thus, the patient had a definite clinical diagnosis of HHT. Genetic testing by next-generation sequencing subsequently identified a heterozygous whole gene deletion of *ENG* (endoglin), encompassing exons 9-15, consistent with an autosomal-dominant pathogenic variant. The variant was classified as pathogenic according to ACMG/AMP (American College of Medical Genetics and Genomics/Association for Molecular Pathology) criteria, confirming HHT type 1.

**Figure 3:**
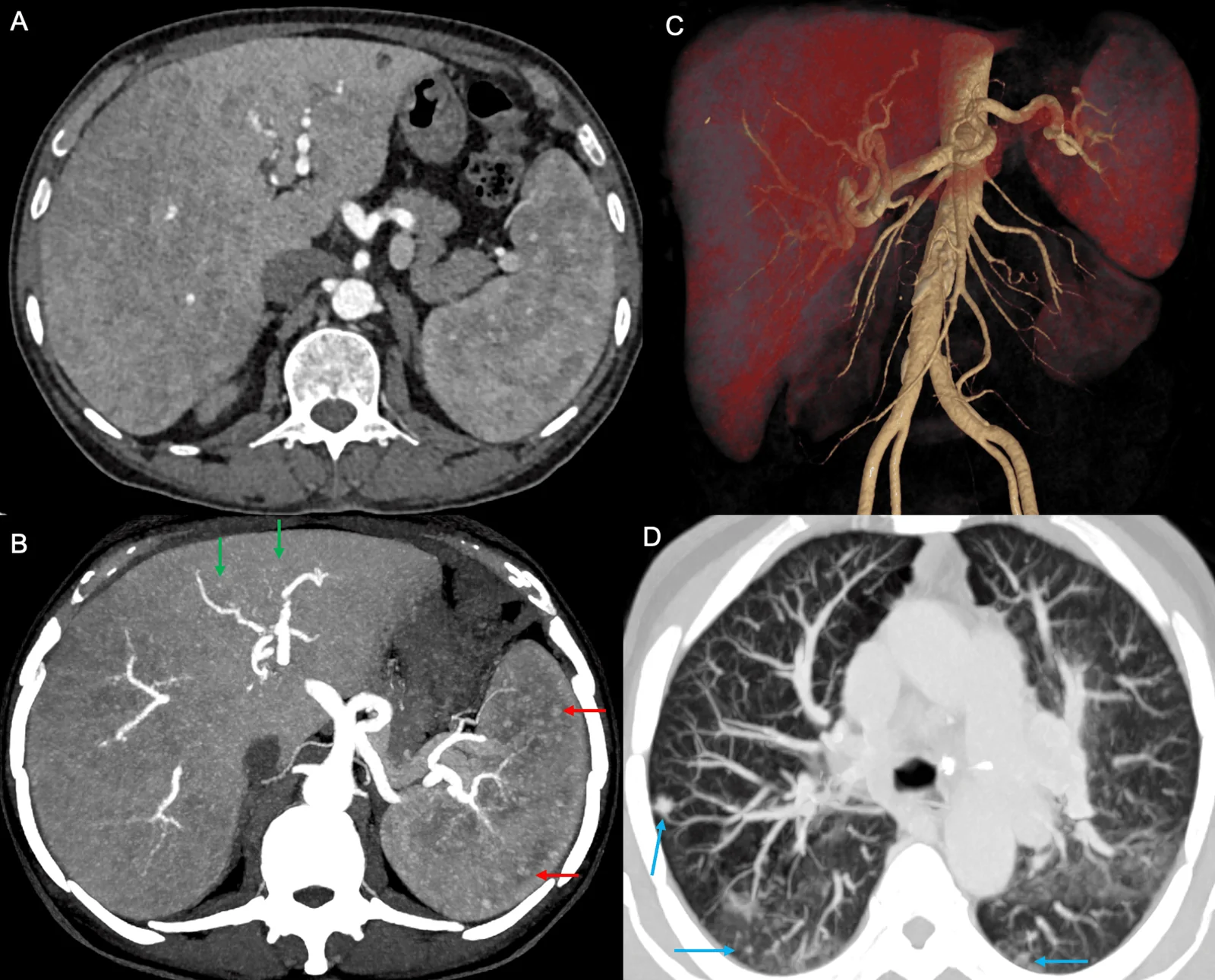
CT angiography imaging in a case of hereditary hemorrhagic telangiectasia. (A) Axial image depicting heterogeneous enhancement in both lobes of the liver. (B) Axial maximum intensity projection (MIP) image showing multiple splenic (few marked with red arrows) and a few hepatic (green arrow) telangiectasias. (C) Volume-rendered image showing marked hepatomegaly and mild-to-moderate splenomegaly. Dilated and tortuous hepatic and splenic arterial branches are seen. Due to shunting, mesenteric arteries are relatively smaller in caliber. (D) Axial MIP image in lung window showing a few pulmonary arteriovenous malformations (blue arrows) in both lungs. Dilated pulmonary artery branches are seen reaching up to the periphery of the lungs.

The combination of characteristic telangiectasias, recurrent epistaxis, visceral AVMs, and a pathogenic *ENG* variant established the diagnosis of definitive HHT. The patient received supportive treatment with PRBC transfusions and intravenous iron replacement for severe iron deficiency anemia. Tranexamic acid was used as an adjunctive hemostatic therapy. Local measures, including endoscopic APC for gastrointestinal lesions and chemical cauterization for nasal telangiectasias, were performed. Despite these interventions and ongoing requirements for blood transfusions and iron supplementation, the patient continued to experience significant recurrent bleeding. Therefore, systemic antiangiogenic therapy with intravenous bevacizumab was initiated. Bevacizumab was administered at a loading dose of 5 mg/kg every two weeks for six doses, followed by 5 mg/kg monthly as maintenance therapy.

Prior to bevacizumab initiation, the patient required PRBC transfusions approximately every 10 days, whereas during the first three months following initiation of bevacizumab, he required only three PRBC transfusions. His ESS improved from 5.7 at baseline to 1.8 after three months of treatment. There was no further melena, and the patient reported an overall improvement in bleeding symptoms. During the first three months of follow-up after bevacizumab initiation, no treatment-related adverse events were reported. Blood pressure and urinary protein levels remained within normal limits, and no thromboembolic events were observed.

## Discussion

HHT is an uncommon inherited vascular disorder with multisystem involvement and considerable clinical heterogeneity. Because manifestations may develop progressively and involve different organ systems, diagnosis can be delayed for many years, particularly when recurrent epistaxis or iron deficiency anemia is managed in isolation. This case highlights the importance of recognizing the underlying vascular disorder in patients with longstanding, otherwise unexplained iron deficiency anemia and recurrent mucosal or gastrointestinal bleeding. In this patient, the prolonged history of epistaxis, mucocutaneous telangiectasias, gastrointestinal bleeding, and visceral AVMs ultimately led to the diagnosis of HHT. The absence of a family history did not exclude the diagnosis, as family disease may remain unrecognized and pathogenic variants may occur de novo [1,2].

The identification of a pathogenic *ENG* variant was particularly relevant. HHT type 1 is associated with pathogenic variants in *ENG*, whereas HHT type 2 is more commonly associated with *ACVRL1* variants. Patients with *ENG* variants have been reported to have a higher prevalence of pulmonary AVMs, making screening for PAVMs particularly important in this subgroup [3]. Pulmonary AVMs create a direct right-to-left shunt that bypasses the pulmonary capillary filter, potentially resulting in hypoxemia and paradoxical embolization of thrombotic or infectious material, with complications that include ischemic stroke and cerebral abscess. Contrast echocardiography is a sensitive screening modality for pulmonary shunting, with a delayed appearance of microbubbles in the left atrium supporting an intrapulmonary rather than an intracardiac shunt. Once a clinically significant PAVM is identified, transcatheter embolization is the preferred preventive intervention [1,2].

Gastrointestinal involvement was an important contributor to this patient's profound chronic iron deficiency. Gastrointestinal telangiectasias are an important cause of iron deficiency and gastrointestinal bleeding, particularly in older individuals with HHT. Endoscopic treatment with APC can control individual bleeding lesions; however, diffuse or recurrent telangiectasias may limit the effectiveness of repeated local therapy [1,2]. In this patient, the requirement for repeated PRBC transfusions despite intravenous iron replacement and local hemostatic measures indicated a substantial ongoing bleeding burden. Management in such patients therefore requires not only correction of iron deficiency but also strategies directed at reducing the underlying vascular bleeding.

Epistaxis is another major contributor to morbidity in HHT. The ESS provides a validated method for quantifying epistaxis severity. In this patient, the baseline ESS of 5.7 indicated a substantial epistaxis burden. Although local measures, including moisturizing therapy, chemical cauterization, laser-based approaches, and other ENT interventions, may provide symptomatic benefit, severe recurrent epistaxis may remain difficult to control [1,2]. The improvement in ESS from 5.7 to 1.8 after three months of bevacizumab in this patient supports a clinically meaningful reduction in epistaxis severity [5].

Systemic bevacizumab has emerged as an important treatment option for severe HHT-associated bleeding. Bevacizumab is a monoclonal antibody targeting vascular endothelial growth factor (VEGF), a key mediator of angiogenesis. Dysregulated angiogenic signaling contributes to the abnormal vascular phenotype of HHT and provides a biological rationale for antiangiogenic therapy [4,6]. In patients with severe bleeding refractory to supportive and local measures, systemic antiangiogenic therapy can therefore be considered.

The largest multicenter observational study of intravenous bevacizumab in HHT, the InHIBIT-Bleed study, evaluated 238 patients across 12 HHT centers. Treatment was associated with a mean increase in hemoglobin of approximately 3.2 g/dL and a mean reduction in ESS of approximately 3.4 points during the first year. Red blood cell transfusion requirements decreased by approximately 82%, while intravenous iron requirements decreased by approximately 70% during the first six months [4]. These findings provide clinical support for the use of systemic bevacizumab in patients with severe HHT-associated bleeding.

In our patient, bevacizumab was administered every two weeks for six doses followed by monthly maintenance therapy. After three months of treatment, hemoglobin increased from 4.1 to 13.1 g/dL, while the requirement for PRBC transfusion decreased from approximately one transfusion every 10 days before treatment to three transfusion episodes during the subsequent three months. The ESS decreased from 5.7 to 1.8, and there was no further melena. These findings indicate improvement in both hematological parameters and clinically overt bleeding following systemic therapy. During the first three months of treatment, blood pressure and urine protein remained within normal limits, and no thromboembolic events were observed.

More recently, randomized clinical trial data have provided additional evidence supporting intravenous bevacizumab in severe HHT-associated bleeding. A randomized phase II study using 5 mg/kg every 14 days for six administrations demonstrated improvement in hemoglobin and a numerical reduction in transfusion requirements, although the trial was underpowered for its primary endpoint [7]. These findings complement the larger observational evidence base and support the role of bevacizumab in appropriately selected patients with severe HHT-associated bleeding.

This case also emphasizes the importance of a multidisciplinary approach to HHT. Optimal management may require coordination between hematology, gastroenterology, interventional radiology, pulmonology, and otorhinolaryngology. In this patient, management included correction of iron deficiency, transfusion support, endoscopic treatment of gastrointestinal bleeding, control of epistaxis, embolization of PAVM, genetic confirmation, and systemic antiangiogenic therapy. Early recognition of HHT in patients with recurrent epistaxis and unexplained iron deficiency may facilitate timely screening for visceral AVMs and appropriate multidisciplinary intervention, potentially reducing cumulative bleeding-related morbidity.

## Conclusions

HHT should be considered in patients with longstanding or refractory iron deficiency anemia, particularly when accompanied by recurrent epistaxis, characteristic mucocutaneous telangiectasias, or gastrointestinal bleeding. The diagnosis may be overlooked in the geriatric population, in whom chronic anemia or recurrent bleeding may be attributed to more common causes. Recognition of the characteristic multisystem manifestations is important because pulmonary and other visceral AVMs may result in serious but potentially preventable complications. Genetic confirmation can establish the diagnosis and facilitate appropriate family evaluation and screening for associated vascular malformations.

In patients with severe HHT-associated bleeding who remain dependent on transfusions and intravenous iron despite local hemostatic measures, systemic bevacizumab is an important therapeutic option. In this patient, a multidisciplinary, multimodal approach comprising PAVM embolization, endoscopic and ENT interventions, iron and transfusion support, and systemic bevacizumab resulted in improvement in hemoglobin, reduced transfusion requirements, improvement in epistaxis severity, and cessation of melena. This case underscores the importance of maintaining a high index of suspicion for HHT, particularly in the geriatric population with otherwise unexplained refractory iron deficiency anemia, and highlights the value of early diagnosis and coordinated multidisciplinary management in reducing disease-related morbidity.
